# In Silico Structural Dynamics and Downstream Bioprocess of Recombinant Human SUMO-IL-33

**DOI:** 10.3390/biom16101451

**Published:** 2026-10-04

**Authors:** Kim-Hung Nguyen, Tan Thanh Mai, Thua-Phong Lam, Long-Hung Dinh Pham, Hoang-Han Do, Minh-Thanh Quach, Khac-Minh Thai, Minh-Quan Le, Quoc-Thai Nguyen

**Affiliations:** 1 School of Pharmacy, University of Medicine and Pharmacy at Ho Chi Minh City, Ho Chi Minh City 700000, Vietnam; nkhung192@gmail.com (K.-H.N.); mthtan@ump.edu.vn (T.T.M.); thua-phong.lam.3910@student.uu.se (T.-P.L.); l.pham23@imperial.ac.uk (L.-H.D.P.); dhhan@ump.edu.vn (H.-H.D.); minh-thanh.quach@universite-paris-saclay.fr (M.-T.Q.); 2 Department of Cell and Molecular Biology, Uppsala University, 75124 Uppsala, Sweden; 3 Department of Chemistry, Imperial College London, London SW7 2AZ, UK; 4 University of Health Sciences, Vietnam National University Ho Chi Minh City, Ho Chi Minh City 700000, Vietnam; tkminh@uhsvnu.edu.vn; 5 Research Center for Discovery and Development of Healthcare Products, Vietnam National University Ho Chi Minh City, Ho Chi Minh City 700000, Vietnam

**Keywords:** IL-33, SUMO-IL-33, 3D structure, receptor

## Abstract

Interleukin-33 (IL-33) is a therapeutic target in inflammatory diseases, yet inhibitor screening is hindered by high commercial protein costs. While expressing IL-33 as a SUMO fusion protein overcomes yield limitations in *Escherichia coli*, whether this intact chimera can directly serve as a screening surrogate remains unresolved. Here, we combined in silico simulations with empirical bioprocess optimization to establish a high-yield pipeline for native human IL-33 production. AI-based structure predictions (AlphaFold and RoseTTAFold) revealed distinct inter-domain dynamics mediated by a flexible linker. Molecular dynamics simulations of the compact RoseTTAFold model suggested potential steric occlusion near receptor-binding interfaces, whereas AlphaFold models exhibited extended conformations, highlighting model-dependent variations that warrant further structural verification. To restore accessibility, tag cleavage was optimized using 1.0% (mol/mol) SUMO protease at 4 °C for 1 h, achieving near-complete digestion. Sandwich ELISA demonstrated an 87.6% molar epitope recognition recovery for cleaved IL-33 versus only 12.0% for the intact fusion, providing empirical evidence of substantial functional epitope masking by the SUMO tag. Two-step IMAC purification yielded ~19% mass recovery (~32.6% molar recovery). These findings demonstrate that tag removal is operationally essential for IL-33 functional availability in screening applications.

## 1. Introduction

Over the past decade, interleukin-33 (IL-33), a pivotal cytokine belonging to the IL-1 family, has emerged as a critical regulator of immune response, demonstrating strong pathological associations with chronic inflammatory conditions (such as asthma, chronic obstructive pulmonary disease, and inflammatory bowel disease) and devastating autoimmune pathologies (including rheumatoid arthritis, systemic lupus erythematosus, and multiple sclerosis) [1,2,3,4]. Structurally characterized by a typical β-trefoil fold, mature IL-33 exerts its biological activity by binding with high affinity to its cognate transmembrane receptor, ST2 (IL-1RL1), initiating a downstream signaling cascade that recruits the IL-1 receptor accessory protein (IL-1RAcP) [5,6]. Recent clinical evidence highlighting the involvement of IL-33 in the aggressive cytokine storms of young COVID-19 patients has further intensified international efforts to develop small-molecule inhibitors or biological blockades targeting the IL-33/ST2 axis [7,8].

Despite widespread therapeutic interest, no small-molecule or biological agents targeting the IL-33/ST2 axis have yet achieved full regulatory approval for clinical use. While several monoclonal antibodies (e.g., tozorakimab and itepekimab) have advanced into or completed Phase III clinical trials, small-molecule drug discovery remains largely in the preclinical phase due to the technical challenge of disrupting broad, shallow protein–protein interaction (PPI) interfaces [9]. To address this hurdle, computational screening initiatives have successfully identified potential lead small molecules capable of targeting the IL-33/ST2 interface in silico [10,11].

To experimentally validate such candidate molecules, commercial screening kits based on sandwich ELISA principles are accessible for target quantification and epitope profiling [12]. However, relying solely on commercial kits for extensive library screening remains cost-prohibitive, limiting high-throughput capacity. Furthermore, while microplate screening kits are convenient for binary endpoint measurements, they cannot provide the bulk quantities of soluble target protein required for custom biophysical and physicochemical characterization methods, such as isothermal titration calorimetry or surface plasmon resonance [5]. Consequently, establishing a robust, reproducible, and cost-effective bioprocess to produce recombinant human IL-33 in-house is an essential step to lower screening overheads and catalyze early-stage pharmaceutical research.

To optimize biosynthetic performance under standard laboratory conditions, direct non-tagged expression of human interleukin-33 (IL-33) in *Escherichia coli* often faces major technical hurdles, including high susceptibility to intracellular proteolysis, insoluble inclusion body formation, and incomplete N-terminal methionine cleavage that compromises protein homogeneity. To overcome these constraints, the pET-SUMO vector system was selected as an established expression platform [13]. The Small Ubiquitin-like Modifier (SUMO) tag (specifically the Smt3 protein derived from *Saccharomyces cerevisiae*) functions as a molecular chaperone that enhances correct polypeptide folding, significantly increases aqueous solubility, and shields the recombinant cytokine from host proteolytic degradation [14]. Crucially, unlike conventional affinity tags, the specific recognition of SUMO by Ulp1 protease (also originating from *S. cerevisiae*) is designed to facilitate cleavage at the C-terminal Gly-Gly junction, aiming to restore the native N-terminus of IL-33 without leaving unwanted scar residues [15]. This structural precision is relevant, as native human IL-33 binds its primary receptor, ST2, with high affinity (*K*_d_ = 0.744 nM) mediated across two distinct binding sites comprising tightly packed, oppositely charged amino acid clusters (PDB ID: 4KC3) [16].

If the intact SUMO-IL-33 chimeric framework could directly bind the ST2 receptor with equivalent spatial orientation and structural affinity, it could function as a more economical, streamlined surrogate in primary screening assays without needing tag cleavage. However, adding a bulky fusion tag risks altering the overall surface topology of the cytokine, potentially masking critical receptor-binding interfaces or epitope regions. To liberate active, mature native IL-33 from the fusion construct, the primary translation product must undergo site-specific enzymatic hydrolysis by SUMO protease, which cleaves the junction amide bond without leaving non-native amino acids at the N-terminus. Yet, adding this secondary enzymatic treatment along with subsequent chromatographic clearance loops inherently compromises the cumulative isolation yield and increases downstream processing complexity.

To evaluate the structural influence of the N-terminal tag and optimize protein recovery, this study combines computational modeling with empirical bioprocess analysis. Using protein structural prediction, molecular dynamics simulations, and protein–protein docking, we explored the spatial orientation of the SUMO moiety relative to the IL-33 core and its potential interference with the ST2-binding interface. Guided by these structural insights, we subsequently optimized the enzymatic cleavage conditions and monitored epitope availability using sandwich ELISA. This integrated workflow clarifies fusion-tag interactions and provides an accessible, cost-effective protocol for producing recombinant human IL-33 suitable for preliminary screening applications.

## 2. Materials and Methods

### 2.1. Methods for Modeling the Three-Dimensional Structure of Proteins

To model the 3D structure of SUMO-IL-33, both template-based and deep learning approaches were employed. Homology modeling was performed using I-TASSER [17,18,19] and C-I-TASSER [17]. For deep learning-based prediction, AlphaFold2 [20] via Google ColabFold [21] and RoseTTAFold [22] were used. The full-length SUMO-IL-33 sequence provided to all tools was as follows (**bold**: SUMO tag; *italics*: native human IL-33):

**MHHHHHHHHKLMSDSEVNQEAKPEVKPEVKPETHINLKVSDGSSEIFFKIKKTTPLRRLMEAFAKRQGKEMDSLRFLYDGIRIQADQTPEDLDMEDNDIIEAHREQIGGG** *SITGISPITEYLASLSTYNDQSITFALEDESYEIYVEDLKKDEKKDKVLLSYYESQHPSNESGDGVDGKMLMVTLSPTKDFWLHANNKEHSVELHKCEKPLPDQAFFVLHNMHSNCVSFECKTDPGVFIGVKDNHLALIKVDSSENLCTENILFKLSET*

### 2.2. Evaluation and Selection of the Best Three-Dimensional Structure of SUMO-IL-33 Built by Different Methods

Each tool generated five structural models. Primary models from I-TASSER and C-I-TASSER were evaluated using C-score, TM-score, and RMSD relative to templates. Models from AlphaFold2 and RoseTTAFold were assessed via pLDDT scores and per-residue error estimates, respectively.

The top model from each program was further validated using Ramachandran plot analysis and the MolProbity server [23]. Key validation criteria included the percentage of residues in favored/allowed regions, clash scores, rotamer quality, and overall MolProbity scores to select the optimal structure for downstream analysis.

### 2.3. Evaluation of the Applicability of SUMO-IL-33 for Biological Assays Compared with Native IL-33 by Molecular Dynamics Simulation and Protein-Protein Docking

The optimal SUMO-IL-33 model was structural aligned with experimental native IL-33 using MOE 2022.02 to evaluate backbone RMSD and steric exposure of receptor-binding sites.

To assess dynamic behavior under physiological conditions, 200 ns molecular dynamics (MD) simulations were performed using GROMACS 2021 with the CHARMM27 force field, TIP3P water model, and 0.15 M NaCl. Energy minimization and 100 ps NVT/NPT equilibrations preceded production runs. Conformational stability and compactness were monitored via backbone RMSD, Cα RMSF, solvent-accessible surface area (SASA), and radius of gyration (R_g_).

Finally, protein–protein docking was conducted using HADDOCK Web Server 2.4 [24,25,26]. The docking protocol was validated by re-docking native IL-33 to the ST2 receptor, followed by docking SUMO-IL-33. Binding performance was evaluated using HADDOCK scores, van der Waals and electrostatic energies, and predicted binding free energy (Δ*G*) [27].

### 2.4. Expression and Purification of Recombinant SUMO-IL-33

*Escherichia coli* BL21(DE3) harboring the pET-SUMO-IL-33 vector (GeneScript, Piscataway, NJ, USA) was used as the expression host. The construct contained the codon-optimized sequence for mature human IL-33 (UniProtKB/Swiss-Prot: O95760, residues 112–270) fused with an N-terminal 8× His-SUMO tag to facilitate soluble expression and affinity purification. Recombinant bacteria were cultured in ZYP-5052 auto-induction medium at 25 °C for 40 h with continuous shaking at 200 rpm, previously described [28]. Harvested cell pellets were resuspended in lysis (Buffer A: 50 mM sodium phosphate pH 7.8, 400 mM NaCl, 100 mM KCl, and 10% glycerol) and disrupted via sonication (10 s pulses × 30 cycles, 40% amplitude, 4 °C) using a JY92-IIN ultrasonic homogenizer (Shanghai Huxi Industry Co., Ltd., Shanghai, China). Cell debris and unbroken bacteria were removed by centrifugation at 18,000× *g* (Eppendorf 5415R) for 30 min at 4 °C.

The resulting clarification supernatant was loaded onto a 5 mL HisTrap HP column (GE Healthcare) pre-equilibrated with buffer A. The column was thoroughly washed with buffer A containing 50 mM imidazole to remove non-specifically bound host proteins. Recombinant SUMO-IL-33 was subsequently eluted by buffer A supplemented with 150 mM imidazole [29]. Fractions corresponding to the purified SUMO-IL-33 fusion protein (~32 kDa), confirmed by 12% SDS-PAGE were pooled, desalted and concentrated into buffer A using a 5 mL desalting column (GE Healthcare, Uppsala, Sweden) coupled with a 10 kDa MWCO Amicon Ultra centrifugal filter unit (Millipore, Billerica, MA, USA).

### 2.5. SUMO Tag Cleavage and Purification of Native IL-33

To liberate native human IL-33, the N-terminal 8× His-SUMO tag was enzymatically cleaved using SUMO protease (Invitrogen, Carlsbad, CA, USA). To evaluate cleavage efficiency, parameters including enzyme-to-substrate molar ratios (0.1% and 1.0% mol/mol), incubation temperatures (4 °C, 25 °C, and 37 °C), and reaction times (1, 2, and 3 h) were systematically evaluated in buffer A.

Following cleavage under optimized conditions, the reaction mixture was subjected to a second-pass IMAC purification using a HisTrap HP column. The cleaved, untagged IL-33 was collected in the flow-through fraction owing to its lack of affinity for the nickel resin. Conversely, residual uncleaved SUMO-IL-33 fusion protein, released 8× His-SUMO tags, and His-tagged SUMO protease were retained on the matrix and were subsequently eluted using Buffer A supplemented with 300 mM imidazole.

### 2.6. Protein Quantification

Total protein concentration was estimated using Bradford assay [30] with bovine serum albumin (BSA) as the reference standard. To evaluate specific epitope exposure and spatial accessibility of IL-33 prior to and following tag removal, sandwich ELISA was performed using a commercial Human IL-33 ELISA Kit (Thermo Fisher Scientific, Waltham, MA, USA, Cat# BMS2048) in accordance with the manufacturer’s protocol.

## 3. Results

### 3.1. 3D Structure Model of SUMO-IL-33

#### 3.1.1. Structural Modeling Using Template-Based and Deep Learning Tools

The 3D structure of SUMO–IL-33 was modeled using template-based methods (I-TASSER and C-I-TASSER), with detailed evaluation metrics summarized in Table S1 (Supplementary Material). C-I-TASSER yielded higher structural confidence than I-TASSER due to its neural network-guided template selection and contact prediction. Specifically, the top-ranked C-I-TASSER model (CIT-H1) achieved a higher C-score (−1.73) and TM-score (0.50) than the top I-TASSER model (IT-H1; C-score = −2.87, TM-score = 0.39).

For deep learning-based tools (AlphaFold2 and RoseTTAFold), five structural models were generated for SUMO–IL-33, among which model 1 demonstrated the highest quality. Figure S1A (Supplementary Material) shows the pLDDT plots of the 5 models ranked from 1 to 5, respectively, with the AF-1 model having the lowest error for amino acids. For the RoseTTA tool, the program provides an evaluation chart for the best model (RF-1 model) as shown in Figure S1B, in which the confidence score of the whole model is 0.62.

**Figure 1:**
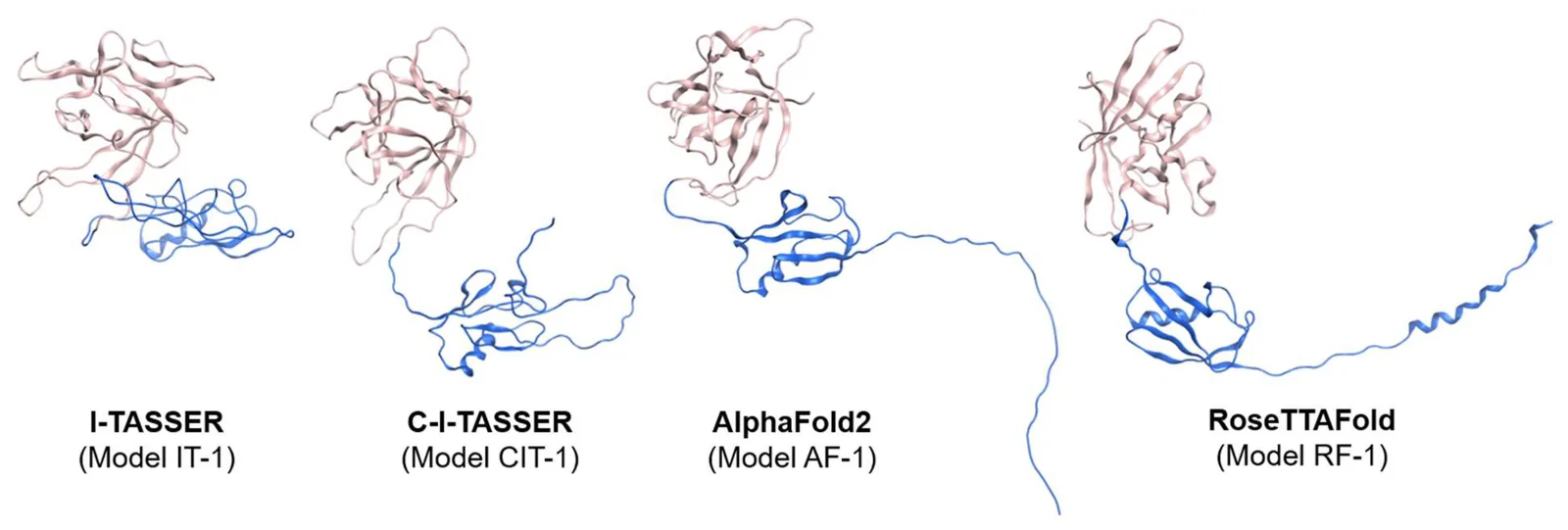
Four best 3D structural models of SUMO-IL-33 were built using different tools. The structure of IL-33 and the SUMO fragment are shown in ribbon form with light pink and blue colors, respectively.

In total, 20 structural models of SUMO–IL-33 were generated using the four computational tools. Based on each tool’s internal scoring system, the best-performing model from each group (CIT-H1, IT-H1, AF-1, and RF-1; Figure 1) was selected for further evaluation. To evaluate local stereochemical quality among candidate models, these four structures were benchmarked using Ramachandran plot analysis and MolProbity clash scores. This served to identify a stereochemically sound starting model (RF-1) for downstream exploratory simulations rather than a definitively optimal global interdomain orientation.

#### 3.1.2. Optimized 3D Structural Model for SUMO-IL-33

Ramachandran plot analysis (Figure S2, Supplementary Material) indicated that the model predicted by RoseTTAFold exhibited superior backbone stereochemistry, with 94.8% of residues in favored regions and 99.3% in allowed regions. Although slightly below ideal theoretical thresholds, the RoseTTAFold model closely approached these quality standards. In contrast, template-based tools (I-TASSER and C-I-TASSER) yielded lower proportions of residues in favored regions (62.3% and 69.8%, respectively). Similarly, AlphaFold2 produced a top model with only 88.1% of residues located in favored regions.

Because backbone geometry alone is insufficient to evaluate overall model plausibility, side-chain rotamers and all-atom steric clashes were further validated using the MolProbity server (Table 1). In terms of clash score alone, AlphaFold2 achieved the best result (clash score = 1.17; 99th percentile). However, considering the composite MolProbity score—which integrates clash score, rotamer outliers, and Ramachandran statistics—the RoseTTAFold model outperformed all other predictions (MolProbity score = 1.60; 92nd percentile).

Evaluating AlphaFold3 [31] showed no noticeable improvement over AlphaFold2 or RoseTTAFold. As shown in Figure S3 (Supplementary Material), the top AlphaFold3 model exhibited 88.06% of residues in favored regions, with a clash score of 7.04 and a MolProbity score of 1.99. In addition, local prediction confidence near the flexible linker joining SUMO and IL-33 (Glu105–Ser111) was closely evaluated (Figure S1). All five AlphaFold2 models predicted these linker residues with low pLDDT scores, comparable to the flexible His-tag and unstructured loop regions of IL-33 (Figure S1A). Conversely, the top RoseTTAFold model assigned significantly lower estimated error (higher reliability) to the corresponding linker residues relative to the His-tag and loop regions (Figure S1B).

Consequently, based on local stereochemical validation from Ramachandran plots and MolProbity scores, the 3D model generated by RoseTTAFold was selected as a stereochemically favorable initial model for downstream exploratory dynamic simulations, rather than a definitively fixed global conformation.

### 3.2. Investigate the Effect of SUMO Fragment on Binding Sites of IL-33 to the ST2 Receptor

#### 3.2.1. Comparison of the SUMO-IL-33 Structure from the RF-1 Model with the Crystal Structure of Native IL-33

**Figure 2:**
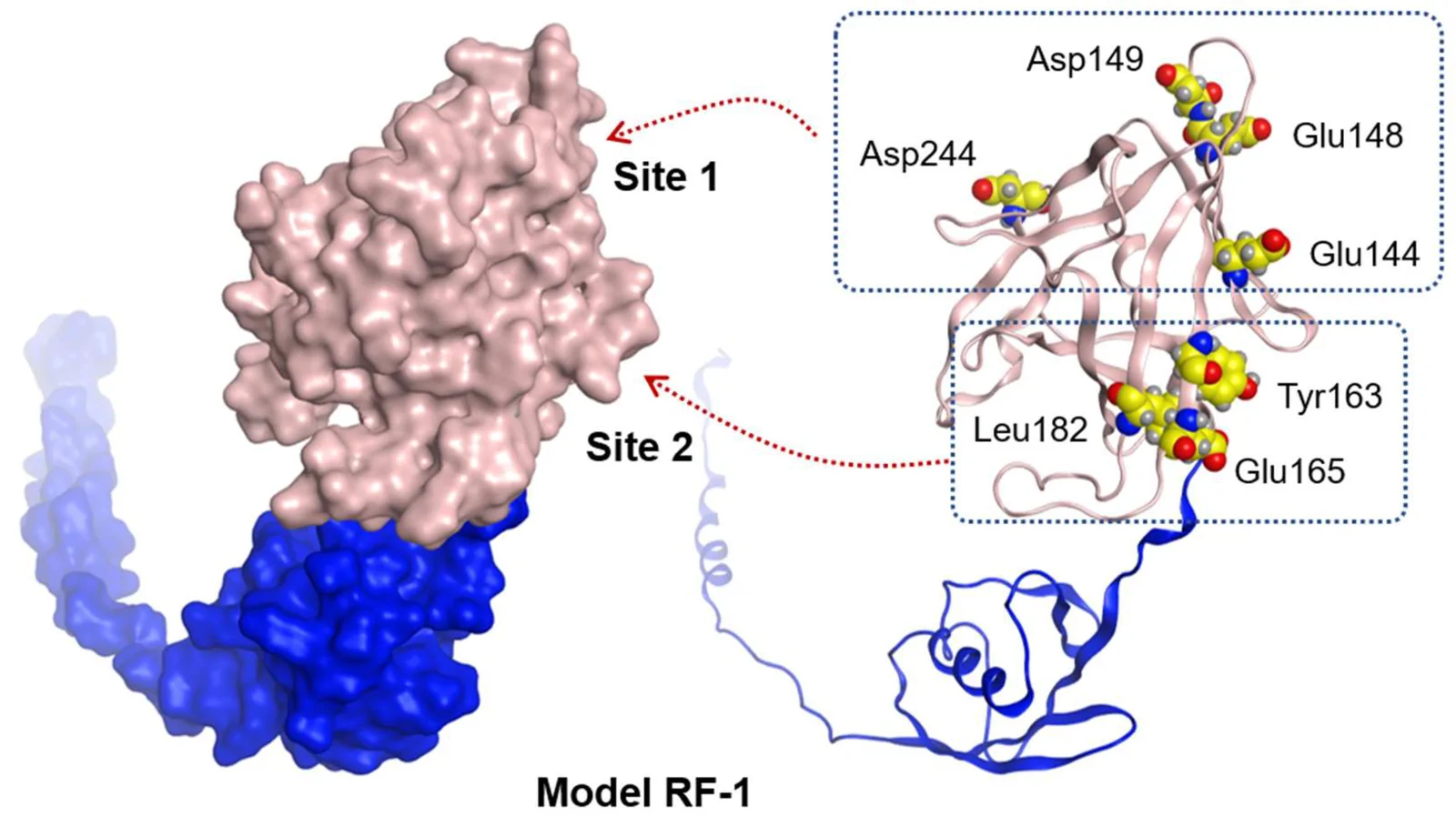
Two binding sites of SUMO-IL-33 (identified by key residues) are not obscured by the SUMO fragment structure. IL-33 is depicted in salmon as surface and ribbon representations, respectively, while the SUMO domain is shown in corresponding representations in blue. On the ribbon structure, hot-spot residues at each receptor-binding site of IL-33 are highlighted in rounded rectangles, with arrows pointing to their corresponding locations on the surface representation.

In the experimentally determined structure, IL-33 binds to its ST2 receptor via two key sites: Site 1 includes important residues demonstrated by Liu et al. via mutagenesis (Glu144, Glu148, Asp149, and Asp244), while site 2 comprises Tyr163, Glu165, and Leu182 [16]. The structure of SUMO-IL-33 from the top model RF-1 using MOE 2022.02 software (Figure 2) shows that neither binding site on IL-33 is masked by the SUMO tag. These static modeling observations reflect a single unperturbed conformation where receptor-binding interfaces remain exposed. However, because static predictions cannot capture linker-mediated flexibility or potential domain reorientations in solution, dynamic molecular simulations are required to evaluate conformational behavior over time.

#### 3.2.2. Structural Evaluation of SUMO–IL-33 Structure Through Molecular Dynamics Simulations

**Figure 3:**
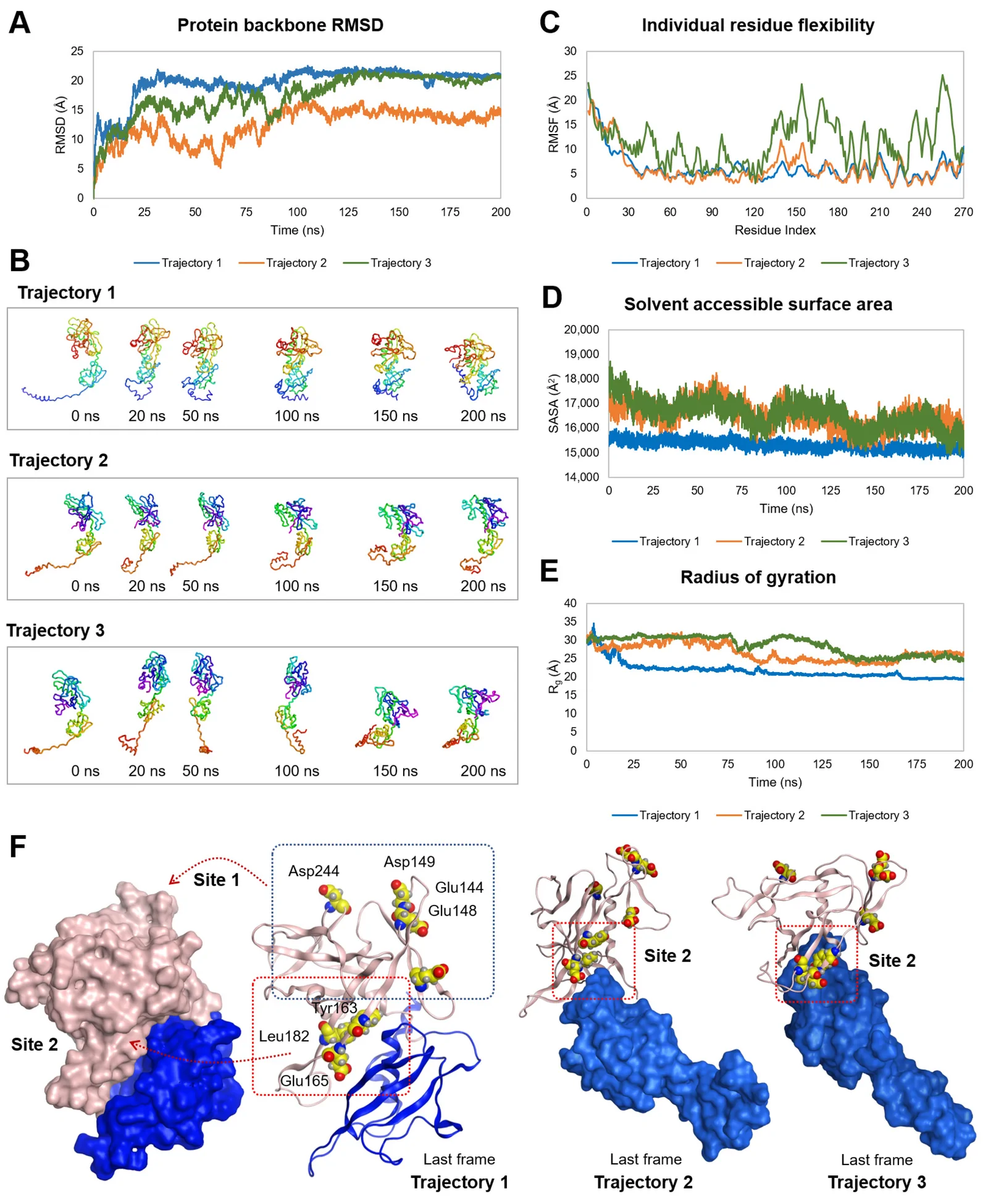
Flexibility analysis of SUMO-IL-33 across three independent 200 ns molecular dynamics simulation trajectories. (**A**) Root-mean-square deviation (RMSD) profiles calculated for backbone C-alpha atoms of the fusion protein. (**B**) Representative conformations of SUMO-IL-33 captured at selected time points. The proteins are colored in a rainbow scheme. (**C**) Root-mean-square fluctuation (RMSF) values reflecting the local flexibility of individual amino acid residues. (**D**) Solvent-accessible surface area (SASA) trajectories indicating time-dependent variations in the solvent-exposed surface of the complex. (**E**) Radius of gyration (R_g_) profiles depicting the overall compactness of the complex over time. (**F**) Terminal structural snapshots of SUMO-IL-33 from each MD trajectory, highlighting the structural impact of the SUMO domain on site 2 of IL-33, a critical region involved in ST2 receptor binding. IL-33 is depicted in salmon as surface and ribbon representations, respectively, while the SUMO domain is shown in corresponding representations in blue. On the ribbon structure, hot-spot residues at receptor-binding site 1 of IL-33 are highlighted in blue rounded rectangles, whereas those at site 2 are framed in red rounded rectangles. Arrows point to the corresponding locations of these residues on the surface representation of SUMO-IL-33.

To further evaluate the dynamic conformation of SUMO-IL-33 under near-physiological conditions, the RF-1 model was subjected to MD simulations for 200 ns across three independent trajectories. Focusing on trajectory 1, results in Figure 3A indicate that after ~20 ns, SUMO-IL-33 underwent a large conformational change, corresponding to an RMSD increase from ~12 Å to ~20 Å. This shift was driven by the folding of the N-terminal SUMO segment from Met1 to Thr33. Beyond 20 ns, the SUMO domain continued folding until reaching equilibrium towards the end of the simulation. Representative SUMO-IL-33 conformations at 0, 20, 100, 150, and 200 ns from trajectory 1 are illustrated in Figure 3B. Based on the RMSF plot in Figure 3C, residues showing large fluctuations were identified as residues 1–33 at the N-terminus of SUMO. The folding of the SUMO domain increased structural compactness, with solvent contact surface area (SASA) and the radius of gyration (R_g_) decreasing over time (as shown in Figure 3D,E). Ultimately, this 200 ns snapshot from RF-1 trajectory 1 was intentionally selected as a representative model of the compact/shielded state to explore potential steric hindrance scenarios in downstream analyses, rather than representing the sole solution conformation.

The SUMO-IL-33 structure at 200 ns in the trajectory 1 was extracted and compared alongside other trajectories in Figure 3F. Re-evaluating this 200 ns snapshot via Ramachandran plot and MolProbity analysis demonstrated high quality, with 94.4% of residues in favored regions and 98.1% in allowed regions. The clash score is 0.48 (99th percentile) and the MolProbity score was 1.36 (98th percentile). Compared with the original RF-1 structure model in Table 1, the structure from the 200 ns MD simulation showed significantly improved stereochemistry. Representation of this structure using MOE software indicated potential spatial hindrance near site 2 in this specific snapshot, whereas site 1 remained unshielded.

Two additional independent simulations initiated with different random initial velocity seeds relative to trajectory 1 were performed, with analyses integrated into Figure 3. Across these two trajectories, backbone RMSD profiles indicate that SUMO-IL-33 reached a stable state after 100 ns. Although the final protein conformations did not superimpose identically with trajectory 1 due to inter-domain linker flexibility, a folding event of the SUMO domain toward IL-33 was consistently observed in the second half of trajectories 2 and 3, primarily affecting site 2 accessibility. This compaction between SUMO and IL-33 was further supported by a gradual decline in both SASA and R_g_ values, which stabilized during the 100–200 ns period. The final structures from each trajectory are displayed in Figure 3F. While partial compaction toward site 2 was observed across these RF-1 trajectories, standard force fields (such as CHARMM with TIP3P water) are known to favor compact states in flexible segments, and non-convergent trajectories reflect conformational sampling limits across the flexible linker rather than a single equilibrium state.

To address potential limitations of relying solely on the RoseTTAFold model and to leverage recent advances in AI-based structure prediction, an additional 100 ns MD simulation was performed on the SUMO-IL-33 fusion structure predicted by AlphaFold3, with trajectory analyses presented in Figure S4 (Supplementary Material). The backbone RMSD profile (Figure S4A) exhibited continuous conformational fluctuations between 40 and 100 ns, indicating that the protein remained in a dynamically evolving state without reaching structural convergence within this timeframe. Representative snapshots sampled along the trajectory (Figure S4B) illustrate that the AlphaFold3 model adopted an extended inter-domain orientation, in which the flexible linker held the SUMO tag away from both site 1 and site 2 of IL-33 required for ST2 receptor binding. Crucially, the contrasting behaviors between the compact RF-1 trajectories and the extended AlphaFold3 trajectory demonstrate that computational predictions of the SUMO–IL-33 inter-domain orientation are highly model-dependent and sensitive to initial starting states, highlighting that these simulation results serve as exploratory hypotheses requiring experimental verification.

### 3.3. Evaluation of SUMO-IL-33 Binding to the ST2 Receptor via Protein–Protein Docking

#### 3.3.1. Validation of Protein–Protein Docking Protocol

**Figure 4:**
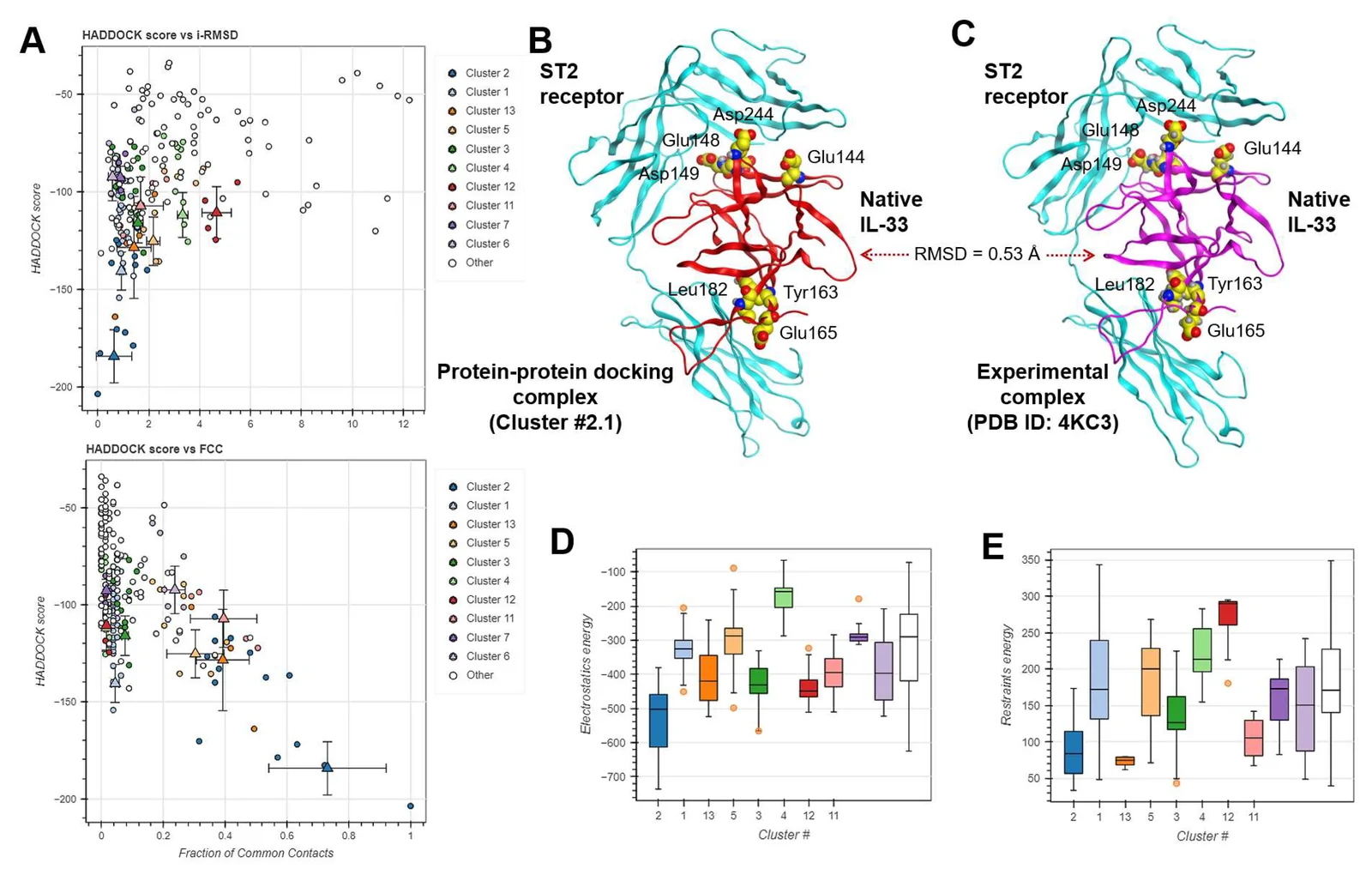
Validation of the protein–protein docking protocol using HADDOCK 2.4 between native IL-33 and the ST2 receptor. (**A**) Scatter plot of HADDOCK score versus interface-RMSD (i-RMSD) and fraction of common contacts (FCC). (**B**) Superimposed 3D structure of the IL-33/ST2 complex predicted by HADDOCK protein–protein docking algorithms. (**C**) Experimental crystal structure of the native IL-33/ST2 complex (PDB ID: 4KC3). (**D**,**E**) Electrostatic energy and restraint violation energy distributions across distinct HADDOCK protein–protein docking clusters. The symbol “#” denotes the cluster number.

First, redocking of intact IL-33 was performed using HADDOCK 2.4 to validate the docking protocol. The redocking process generated 120 IL-33/ST2 structural complexes, categorized into 13 major clusters. Results of the 10 best-performing clusters are presented in Figure 4A, among which cluster 2 achieved the top rank based on the highest HADDOCK score (−184.5 ± 11.8) and the most favorable Z-score (−2.5), containing 14 member structures. The top-ranked structure in cluster 2 is cluster 2.1 as illustrated in Figure 4B. The predicted docking pose closely matched the experimental X-ray crystal structure of the IL-33/ST2 complex (PDB code: 4KC3; Figure 4C), in which IL-33 interacts with ST2 primarily through two distinct sites with key residues highlighted in Figure 4B,C. Structural superposition between the native crystal structure and the docked model yielded a backbone RMSD of only 0.53 Å, confirming high docking accuracy. For the cluster 2 complex, the calculated van der Waals and electrostatic energies were −48.8 ± 8.3 and −673.0 ± 43.5 kcal/mol, respectively (Figure 4D,E). The predicted binding free energies score for native IL-33/ST2 binding were Δ*G* = −14.5 kcal/mol establishing a reference computational baseline for comparison.

#### 3.3.2. Investigation of SUMO-IL-33 Binding to the ST2 Receptor via Protein–Protein Docking

**Figure 5:**
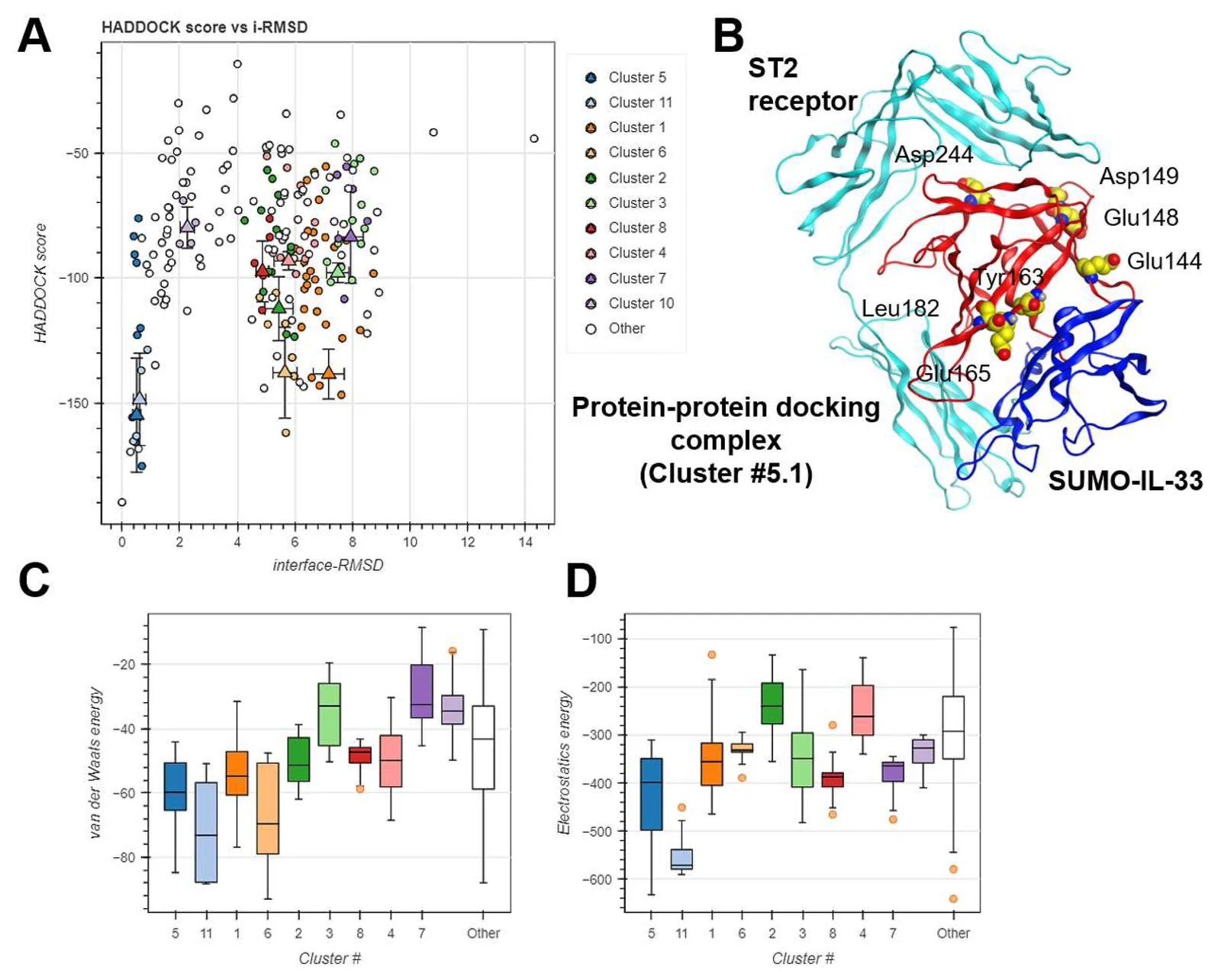
Protein–protein binding modes and interaction characteristics of SUMO-IL-33 bound to the ST2 receptor. (**A**) Correlation plot of HADDOCK score versus interface-RMSD. (**B**) Docked 3D structural model of the ST2/SUMO-IL-33 complex belonging to cluster 5.1, illustrating that IL-33 exclusively engages ST2 at site 1. ST2 is represented as a ribbon in cyan, whereas IL-33 in the fusion protein is shown as a ribbon in red, and the SUMO domain in blue. (**C**,**D**) van der Waals and electrostatic energy contributions evaluated across the predicted HADDOCK clusters. The symbol “#” denotes the cluster number.

MD simulations revealed a diverse conformational flexibility for SUMO-IL-33. To evaluate the interaction between SUMO-IL-33 and the ST2 receptor through protein–protein docking, the end-point snapshot from MD simulation trajectory 1 of the RF-1 model was selected and subjected to HADDOCK 2.4 docking against ST2. The server generated 105 structure complexes divided into 11 clusters, with the top ten clusters presented in Figure 5. Cluster 5 exhibited the highest reliability with a HADDOCK score of −154.9 ± 19.7; a Z-score of −1.5; a van der Waals energy of −71.4 ± 16.8 kcal/mol, and an electrostatic energy of −546.0 ± 55.7 kcal/mol. These parameters suggest that binding of this SUMO–IL-33 conformation to the ST2 receptor is qualitatively less favorable than that of native IL-33, accompanied by a modest reduction in calculated electrostatic interaction energy. Experimental results have demonstrated that IL-33 binds ST2 mainly through salt bridges between negatively charged amino acids (Glu, Asp) and positively charged amino acids (Lys, Arg) [16]. Analysis of cluster 5.1 (the top-ranked structure in cluster 5; Figure 5B) reveals that key site 1 residues (Glu144, Glu148, Asp149) exhibit restricted accessibility to ST2 surface residues compared to the native complex (Figure 4B,C). Additionally, site 2 residues (such as Tyr163, Glu165, Leu182) exhibit partial spatial obstruction in this snapshot due to the proximity of the SUMO domain. Consequently, the predicted binding free energy score for this SUMO–IL-33 complex was Δ*G* = −12.2 kcal/mol. Overall, protein–protein docking results qualitatively reflect a trend toward reduced binding propensity for the intact fusion construct (Δ*G* = −12.2 vs. −14.5 kcal/mol), serving as an exploratory indicator of potential spatial interference near the binding interface rather than a quantitative kinetic measurement.

### 3.4. Enzymatic Removal of the SUMO Tag and Recovery of Native IL-33

**Figure 6:**
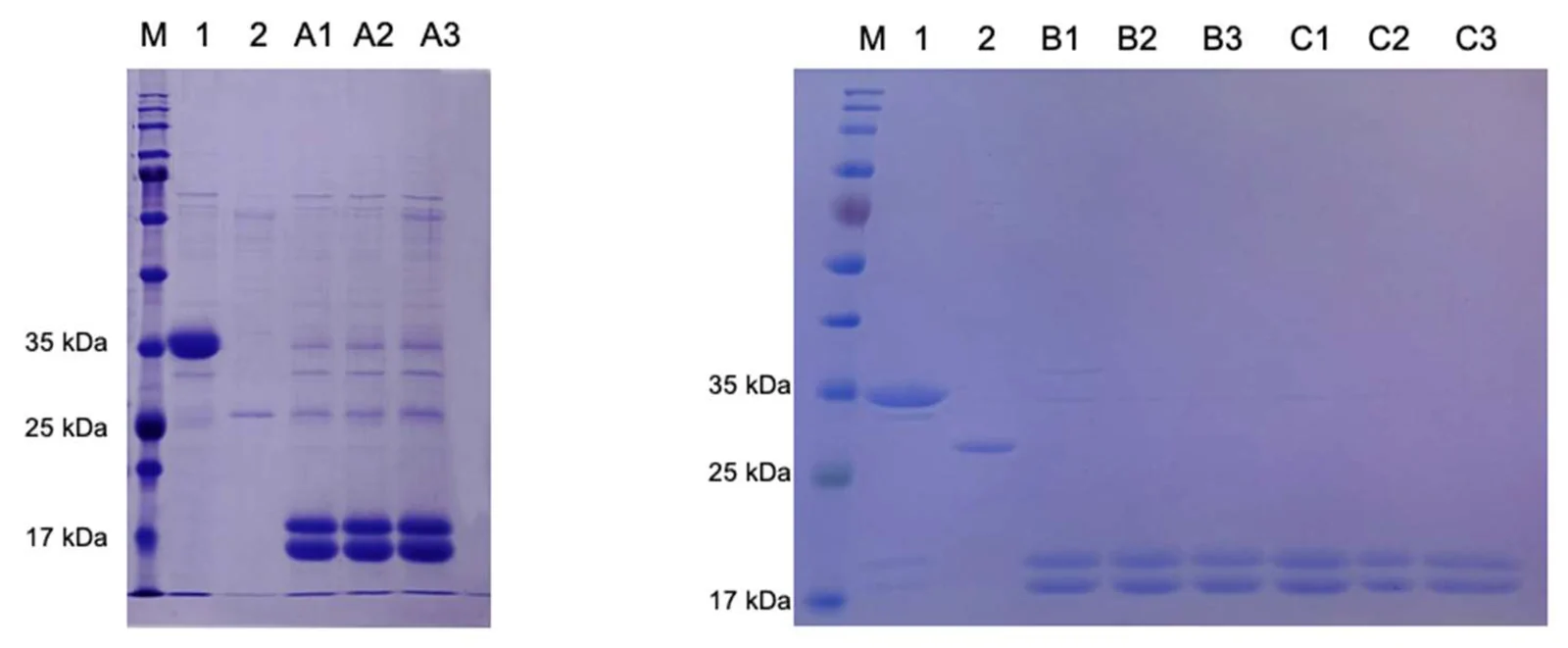
Cleavage reaction of SUMO-IL-33 (lane 1, ~30.8 kDa) at 4 °C (A1–3) or 25 °C (B1–3) and 37 °C (C1–3) by 1% (mol/mol) SUMO protease (lane 2, ~29.2 kDa), at different incubation time from 1 to 3 h (1–3) to obtain native IL-33 (~17.9 kDa) and His-SUMO tag (~12.8 kDa). M: marker.

To obtain native human IL-33 without extraneous N-terminal amino acids, we systematically optimized the enzymatic cleavage of the 8× His-SUMO fusion tag. The cleavage efficiency was highly dependent on the enzyme-to-substrate molar ratio. At a low protease load of 0.1% (mol/mol), the reaction proceeded sluggishly, achieving only partial digestion even after an extended incubation period of 3 h. Conversely, increasing the enzyme load to 1.0% (mol/mol) drastically accelerated the cleavage kinetics, resulting in near-complete fusion protein processing within just 1 h at 4 °C (Figure 6). Notably, elevating the reaction temperature (to 25 °C or 37 °C) or extending the incubation duration provided no discernible increase in cleavage yield, while potentially increasing the risk of non-specific proteolysis or target protein degradation. Consequently, digestion with 1.0% SUMO protease at 4 °C for 1 h was selected as the standardized condition for downstream processing.

**Figure 7:**
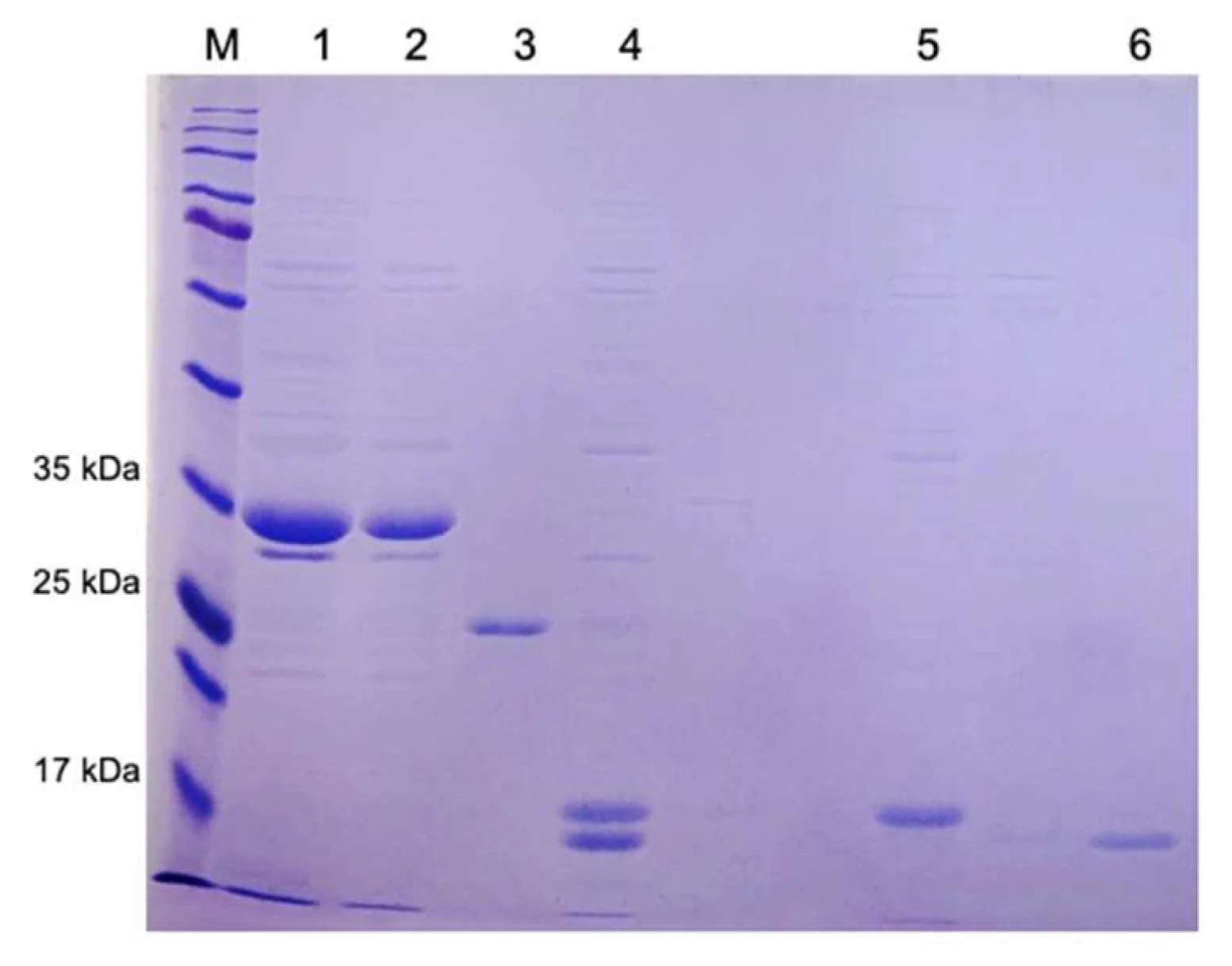
Purification of native IL-33 by 2nd HisTrap. Lanes 1 and 2: SUMO-IL-33 (un- and desalted, respectively, ~30.8 kDa), 3: SUMO protease (~29.2 kDa), 4: cleavage mixture, 5: native IL-33 (~17.9 kDa) 6: His-SUMO tag (~12.8 kDa), M: marker.

The digestion mixture was subsequently resolved via subtractive IMAC purification (Figure 7). The untagged native IL-33 was efficiently recovered in the unbound flow-through fraction, while residual uncleaved fusion protein, liberated SUMO tags, and the His-tagged protease were quantitatively retained on the column matrix. From an initial pool of 203.35 mg desalted SUMO-IL-33 (from 1 L of culture), a total of 38.56 mg of purified native IL-33 was recovered, corresponding to mass recovery yield of ~19% and a molar recovery yield of 32.6% relative to the starting soluble fusion protein input (Figure 7).

### 3.5. Comparative Quantification and Epitope Exposure (ELISA vs. Bradford)

To evaluate the spatial exposure and immunological accessibility of the IL-33 domain, total protein concentrations determined by the Bradford assay were compared on a molar basis with immunologically active concentrations measured via sandwich ELISA (Table 2). To account for the molecular weight disparity between the fusion intermediate (30.8 kDa) and mature native IL-33 (17.9 kDa), input concentrations were converted to molarities. The cleaved native IL-33 construct displayed marked epitope accessibility, with sandwich ELISA recognizing 87.6 ± 3.6% of the total molar protein input. In contrast, the intact SUMO-IL-33 fusion protein exhibited significantly diminished antibody recognition, yielding a molar epitope recovery of only 12.0 ± 0.8%. This ~7-fold discrepancy indicates that the N-terminal 8× His-SUMO domain exerts notable steric hindrance, masking key antigenic epitopes on human IL-33. Enzymatic removal of the SUMO tag successfully relieved this spatial occlusion, substantially restoring the immunological availability of the recombinant cytokine for downstream applications.

## 4. Discussion

The development of cost-effective platforms for producing recombinant human cytokines is essential for expanding drug discovery capabilities, particularly in resource-constrained research environments. In this study, we addressed a critical translational bottleneck in anti-inflammatory drug screening targeting the IL-33/ST2 axis: determining whether the recombinant SUMO-IL-33 fusion protein can directly serve as a suitable molecular target in binding assays or if downstream cleavage is operationally necessary to expose relevant epitopes. By integrating dynamic computational simulations with empirical bioprocess validation, our findings provide a structural explanation for fusion-tag interference and establish an optimized workflow for native cytokine recovery.

Using bioinformatics approaches, multiple 3D structural models of SUMO-IL-33 were constructed using four distinct tools with different algorithms, including I-TASSER [17,18,19], C-I-TASSER [17], AlphaFold2 [20,21], AlphaFold3 [31], and RoseTTAFold [22]. Based on stereochemical evaluation of all-atom clashes and side-chain rotamers via MolProbity [23], AI-based structure prediction tools (AlphaFold and RoseTTAFold) yielded superior models compared to traditional template-based methods. Among these, model RF-1 generated by RoseTTAFold exhibited the highest overall structural quality. While MolProbity evaluates local stereochemistry and rotamer quality [23], it does not measure interdomain orientations across flexible linkers; thus, model RF-1 was selected primarily as a representative initial conformation for downstream exploratory simulations.

Initial static structural predictions generated by RoseTTAFold (model RF-1) suggested that both binding sites 1 and 2 on IL-33 remained exposed despite the presence of the N-terminal SUMO tag. However, explicit-solvent MD simulation (three independent 200 ns trajectories) revealed a fundamental shift in protein conformation. In trajectory 1, a major structural collapse occurred within the first 20 ns at the flexible N-terminal junction (residues Met1–Thr33), driving the SUMO domain onto the cytokine core. This dynamic adaptation significantly reduced both solvent-accessible surface area (SASA) and the radius of gyration (R_g_), yielding a compact, thermodynamically stable state. In this trajectory, this domain motion suggested potential steric occlusion around site 2 (a region involving residues such as Tyr163, Glu165, and Leu182), a region known to be essential for stabilizing the cytokine–receptor complex [16].

The potential functional impact of this steric hindrance was further supported by molecular docking predictions and quantitative immunoassay tracking. HADDOCK protein-protein docking [24,25,26] qualitatively suggested a relative trend toward reduced binding capability for the SUMO-IL-33 chimera (Δ*G* = −12.2 kcal/mol vs. −14.5 kcal/mol for native IL-33). However, given the inherent scoring limitations of rigid/semi-flexible docking algorithms relative to experimental binding kinetics, these values serve as qualitative indicators of potential spatial interference at the interface [16] rather than precise quantitative affinity measurements.

In this study, the top-ranked structural model from RoseTTAFold was selected as the representative starting structure for molecular dynamics simulations and protein–protein docking. Notably, this RoseTTAFold conformation exhibited minimal initial steric occlusion compared to other predicted models (Figure 1), suggesting that the initial structure itself was not inherently biased toward preventing receptor engagement. However, because alternative predicted structures—including the remaining four RoseTTAFold models and all five AlphaFold2 structures (Figure S5)—display distinct inter-domain orientations across the flexible linker, we acknowledge that focusing on a single starting model represents an inherent scope limitation of the current work. A more comprehensive exploration involving these alternative RoseTTAFold and AlphaFold conformations will be required in future work to fully map the conformational landscape and evaluate whether alternative starting states undergo similar domain reorientations.

Although MD simulations were conducted with a cumulative time of 600 ns across independent replicates, the trajectory outcomes remain influenced by the choice of the initial RoseTTAFold model. In particular, linker flexibility significantly modulates the relative domain orientation and interactions between SUMO and IL-33, as observed in the AlphaFold3 simulation trajectory. Therefore, experimental validation remains essential to confirm the reliability of MD simulation findings for the SUMO-IL-33 construct. Additionally, standard force fields (such as CHARMM) paired with TIP3P water models have a known tendency to over-compact flexible segments, and trajectories that do not converge reflect conformational sampling limits rather than a single equilibrium state. To definitively validate these computational models, future experimental characterizations—including Small-Angle X-ray Scattering (SAXS) to measure R_g_ and *P*(r), targeted site-directed mutagenesis of predicted contact residues, and surface plasmon resonance (SPR) kinetics—will be required to discriminate compact from extended ensembles.

While these immunoassay results demonstrate a marked reduction in epitope exposure (12.0% for SUMO-IL-33 vs. 87.6% for cleaved IL-33), sandwich ELISA lacks spatial resolution and cannot differentiate between site-specific occlusion, alternative epitope masking, or generic steric shielding by the 12.8 kDa tag. Nevertheless, the marked reduction in immunorecognition empirically confirms functional steric shielding by the N-terminal SUMO moiety. Together, these operational immunoassay data indicate that intact SUMO-IL-33 is not a suitable direct surrogate for screening applications, making tag removal operationally necessary.

Driven by this structural requirement, we optimized the downstream bioprocess to achieve efficient tag cleavage and target protein recovery. Enzymatic cleavage using 1.0% (mol/mol) SUMO protease at 4 °C for 1 h achieved near-complete digestion while minimizing non-specific degradation. Subsequent subtractive IMAC successfully removed the cleaved 8× His-SUMO tag, residual uncleaved fusion protein, and His-tagged protease, yielding 38.56 mg of native IL-33 from 203.35 mg of initial fusion intermediate. This corresponds to a mass recovery yield of ~19% and a molar recovery yield of ~32.6%, taking into account the removal of the ~12.8 kDa SUMO partner. Overall, this integrated workflow provides a practical and cost-effective strategy for producing recombinant human IL-33, facilitating downstream immunorecognition studies and preliminary inhibitor screening. This localized, high-yield production pipeline is particularly valuable in resource-constrained research environments, such as in Vietnam, where translational initiatives targeting IL-33 face severe practical and financial bottlenecks due to heavy reliance on expensive imported reagents.

## 5. Conclusions

This study presents a combined structural and bioprocess evaluation of recombinant human SUMO-IL-33. Exploratory molecular dynamics simulations and molecular docking suggested that inter-domain flexibility of the SUMO tag may cause spatial hindrance near receptor-binding interfaces. Empirical immunoassay evaluation demonstrated substantial epitope masking by the tag, as intact SUMO-IL-33 exhibited only 12.0% epitope exposure compared to 87.6% for the cleaved form, establishing that tag removal is operationally necessary to restore full accessibility.

To address this tag-induced shielding, an enzymatic cleavage protocol (1.0% mol/mol SUMO protease at 4 °C for 1 h) coupled with subtractive IMAC was established, yielding 38.56 mg of purified human IL-33 (~32.6% molar recovery). This workflow offers an accessible and cost-effective pipeline for recombinant human IL-33 production in Vietnam, providing a viable protein source for future high-throughput screening and biochemical characterization targeting the IL-33/ST2 signaling pathway.

## Figures and Tables

**Table 1:** Evaluation of the best SUMO-IL-33 3D structures obtained from I-TASSER, C-I-TASSER, AlphaFold2, RoseTTAFold tools using MolProbity.

Evaluation Criteria	I-TASSER (Model IT-1)	C-I-TASSER (Model CIT-1)	AlphaFold2 (Model AF-1)	RoseTTAFold (Model RF-1)
Clash score	31.2	26.79	1.17	4.69
Rank conflict by percentile	14/100	18/100	99/100	95/100
Ramachandran favored	62.3%	69.8%	88.1%	94.8%
Ramachandran outliers	13.4%	9.7%	4.1%	0.8%
MolProbity score	3.80	3.56	1.77	1.60
MolProbity score rating by percentile	4/100	8/100	87/100	92/100
Number of Poor-quality links	10/2213 (0.45%)	1/2213 (0.05%)	0/2214 (0.00%)	5/2214 (0.23%)
Number of poor-quality angles	135/2984 (4.54%)	84/2984 (2.82%)	26/2986 (0.87%)	9/2986 (0.30%)

**Table 2:** Comparative evaluation of total protein content and molar epitope accessibility between intact SUMO-IL-33 fusion and cleaved native IL-33.

Sample	Total Protein Bradford (mg/mL)	Molar Input Bradford (µM)	Back-Calculated ELISA (µM) ^a^	Molar Epitope Recovery (%) ^b^
SUMO-IL-33	1.92 ± 0.05	62.3 ± 1.6	7.6 ± 0.4	12 ± 0.8%
Native IL-33	0.95 ± 0.03	53.1 ± 1.7	46.5 ± 1.2	87.6 ± 3.6%

^a^ After accounting for sample dilution factors (4 × 10^6^ for SUMO-IL-33 and 2 × 10^6^ for native IL-33), assuming equivalent MW between the kit standard and mature native IL-33 (17.9 kDa). ^b^ Expressed as (molar ELISA/molar Bradford) × 100%.

## Data Availability

The original contributions presented in this study are included in the article/Supplementary Material. Further inquiries can be directed to the corresponding author(s). The atomic coordinates used in this study were retrieved from the Protein Data Bank under accession code 4KC3. All generated 3D models, molecular dynamics trajectories, and experimental datasets are available from the corresponding author upon reasonable request.

## Supplementary Materials

The following supporting information can be downloaded at https://www.mdpi.com/article/10.3390/biom16101451/s1: Figure S1: Evaluation of the 3D structural models of SUMO-IL-33 predicted by artificial intelligence-based tools; Figure S2: Ramachandran plot analysis of the best 3D structural models of SUMO-IL-33 obtained from I-TASSER (A), C-I-TASSER (B), AlphaFold2 (C), and RoseTTAFold (D); Figure S3: Structural model of SUMO-IL-33 predicted by AlphaFold3; Figure S4: Trajectory analysis of the 100 ns MD simulation for the AlphaFold3-predicted SUMO-IL-33 structure; Figure S5: Structural models of SUMO-IL-33 predicted by RoseTTAFold (A) and AlphaFold2 (B); Table S1: 3D structural models of SUMO-IL-33 obtained by template-based tools I-TASSER and C-I-TASSER.

## Abbreviations

The following abbreviations are used in this manuscript:

IL	Interleukin
IL1RL1	Interleukin-1 receptor-like 1
IMAC	Immobilized metal affinity chromatography
MD	Molecular dynamics
RAcP	Receptor accessory protein
PPI	Protein–protein interaction
SUMO	Small Ubiquitin-like modifier
AI	Artificial intelligence
RMSD	Root-mean-square deviation
RMSF	Root-mean-square fluctuation
pLDDT	Predicted local distance difference test
SASA	Solvent–accessible surface area
R_g_	Radius of gyration
BSA	Bovine serum albumin
