# Supplementary material for: In Silico Structural Dynamics and Downstream Bioprocess of Recombinant Human SUMO-IL-33
Source: Biomolecules. 2026 Oct 4;16(10):1451. doi: 10.3390/biom16101451 (PMC13702225; doi:10.3390/biom16101451)
Supplement: Supplementary file 1 [file biomolecules-16-01451-s001.zip › biomolecules-4512098-supplementary.pdf]

biomolecules

MDPI

Table S1. 3D structural models of SUMO-IL-33 obtained by template-based tools I-TASSER and C-I-TASSER.

Homology modeling tools	Obtained models	C-score	RMSD (Å)	TM-score
I-TASSER	IT-H1	−2.87	12.8 ± 4.2	0.39 ± 0.13
	IT-H2	−3.07		
	IT-H3	−3.54		
	IT-H4	−3.65		
	IT-H5	−3.76		
C-I-TASSER	CIT-H1	−1.73	9.9 ± 4.6	0.50 ± 0.15
	CIT-H2	−2.23		
	CIT-H3	−3.76		
	CIT-H4	−3.76		
	CIT-H5	−3.76		

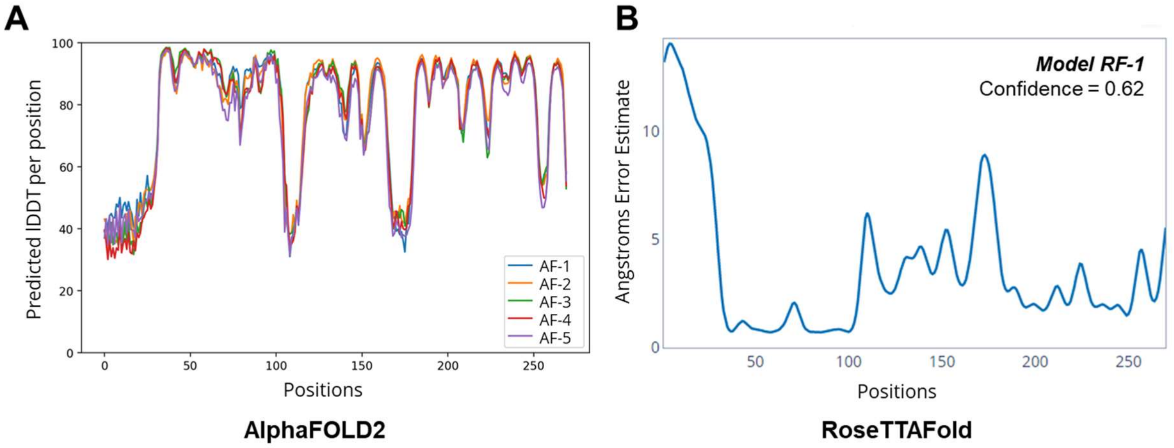

Figure S1. Evaluation of the 3D structural models of SUMO-IL-33 predicted by artificial intelligence-based tools. (A) Predicted Local Distance Difference Test (pLDDT) plot evaluating five structural models generated by AlphaFold2. (B) Predicted Aligned Error (PAE) plot assessing the top structural model obtained from RoseTTAFold.

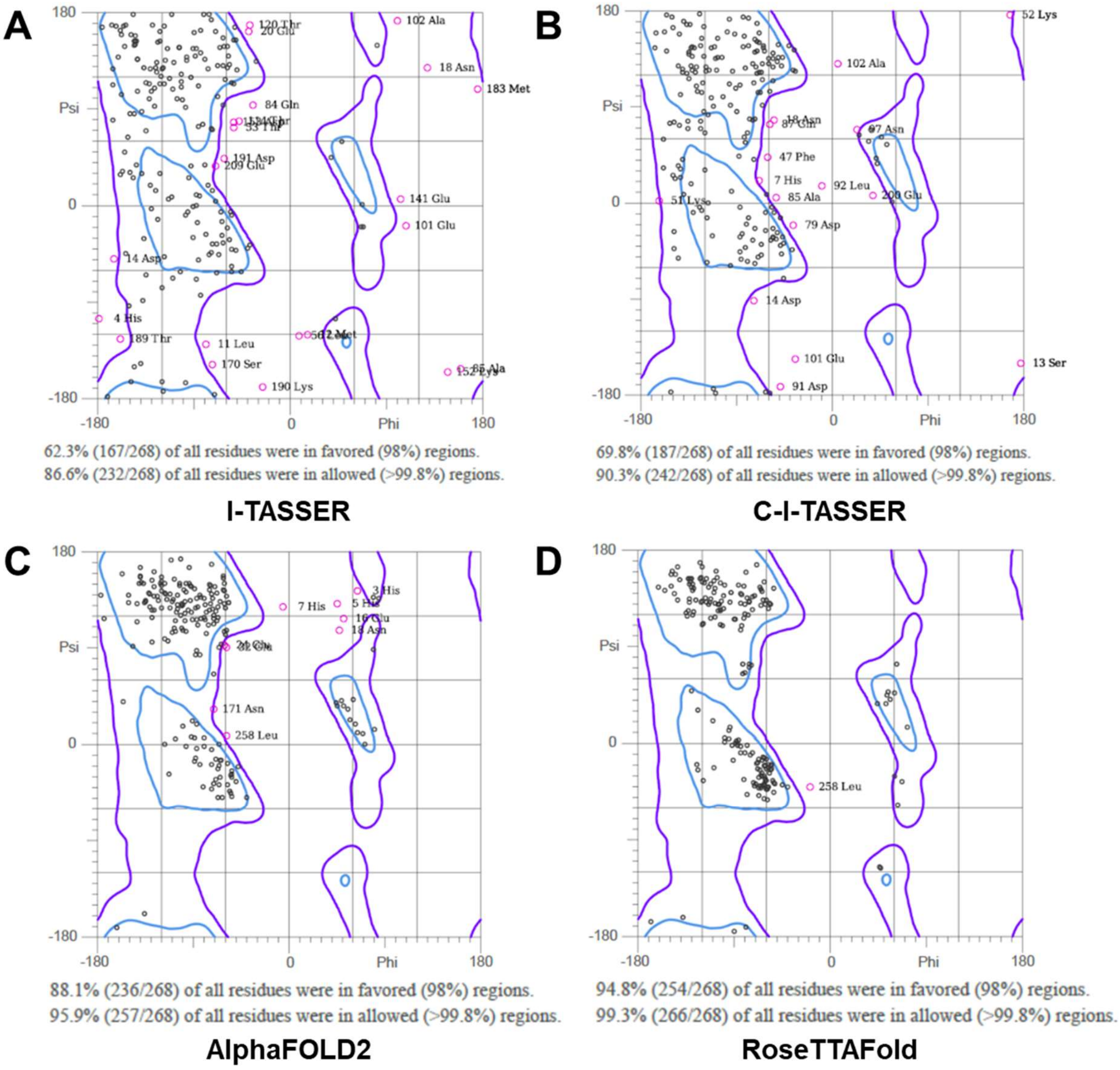

Figure S2. Ramachandran plot analysis of the best 3D structural models of SUMO-IL-33 obtained from I-TASSER (A), C-I-TASSER (B), AlphaFold2 (C), and RoseTTAFold (D).

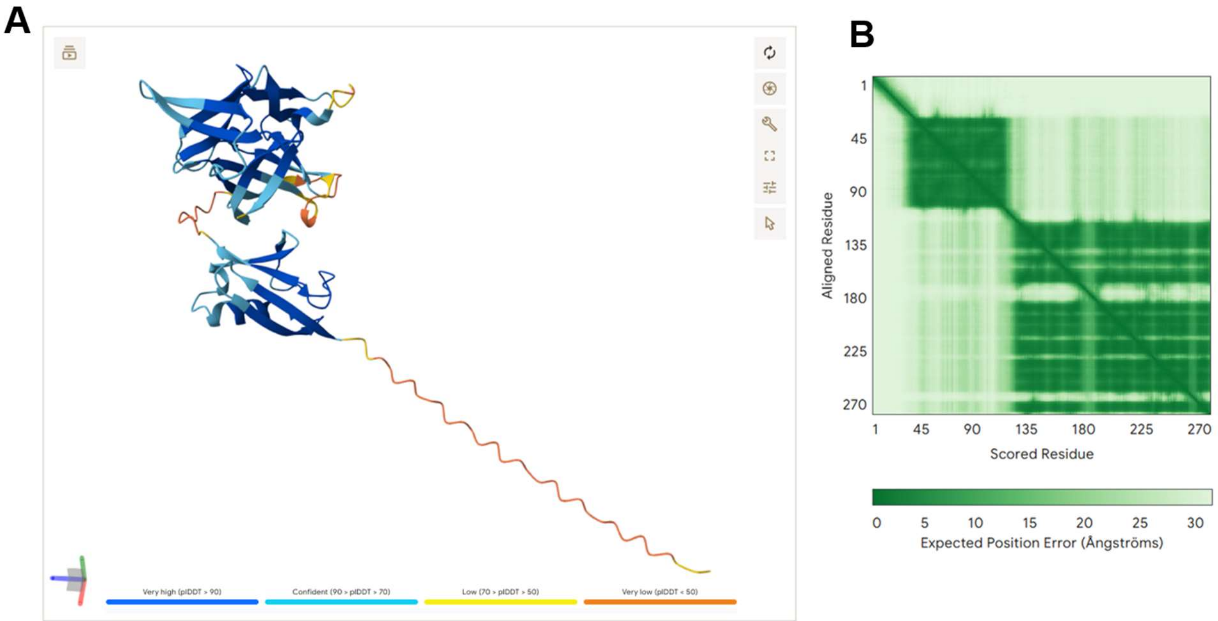

C

Clashscore, all atoms:	7.04		86th percentile* (N=1784, all resolutions)
Clashscore is the number of serious steric overlaps (> 0.4 Å) per 1000 atoms.			
Poor rotamers	0	0.00%	Goal: <0.3%
Favored rotamers	248	100.00%	Goal: >98%
Ramachandran outliers	6	2.24%	Goal: <0.05%
Ramachandran favored	236	88.06%	Goal: >98%
Rama distribution Z-score	-0.58 ± 0.49		Goal: abs(Z score) < 2
MolProbity score^	1.99		76th percentile* (N=27675, 0Å - 99Å)
Cβ deviations >0.25Å	0	0.00%	Goal: 0
Bad bonds:	0 / 2214	0.00%	Goal: 0%
Bad angles:	4 / 2986	0.13%	Goal: <0.1%
Cis Prolines:	1 / 11	9.09%	Expected: ≤1 per chain, or ≤5%
CaBLAM outliers	0	0.0%	Goal: <1.0%
CA Geometry outliers	1	0.38%	Goal: <0.5%
Chiral volume outliers	0/325		
Waters with clashes	0/0	0.00%	See UnDowser table for details

Figure S3. Structural model of SUMO-IL-33 predicted by AlphaFold3. (A) 3D structure colored by pLDDT score. (B) Per-residue predicted aligned error (PAE). (C) Model evaluation results from the MolProbity server.

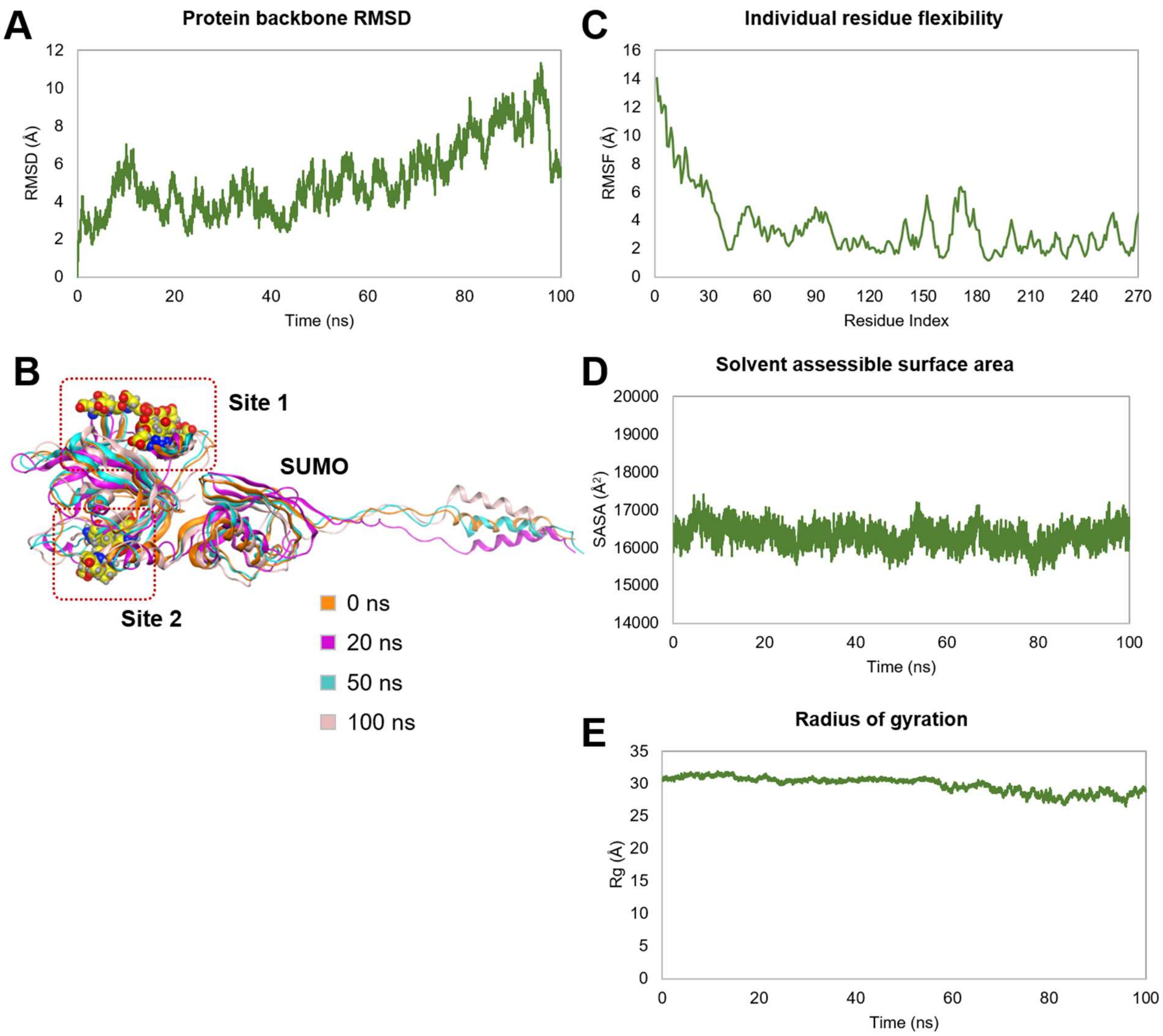

Figure S4. Trajectory analysis of the 100 ns MD simulation for the AlphaFold3-predicted SUMO-IL-33 structure. (A) Root-mean-square deviation (RMSD) profiles calculated for backbone C-alpha atoms of the fusion protein. (B) Representative conformations of SUMO-IL-33 captured at selected time points. (C) Root-mean-square fluctuation (RMSF) values reflecting the local flexibility of individual amino acid residues. (D) Solvent-accessible surface area (SASA) trajectories indicating time-dependent variations in the solvent-exposed surface of the complex. (E) Radius of gyration (Rg) profiles depicting the overall compactness of the complex over time

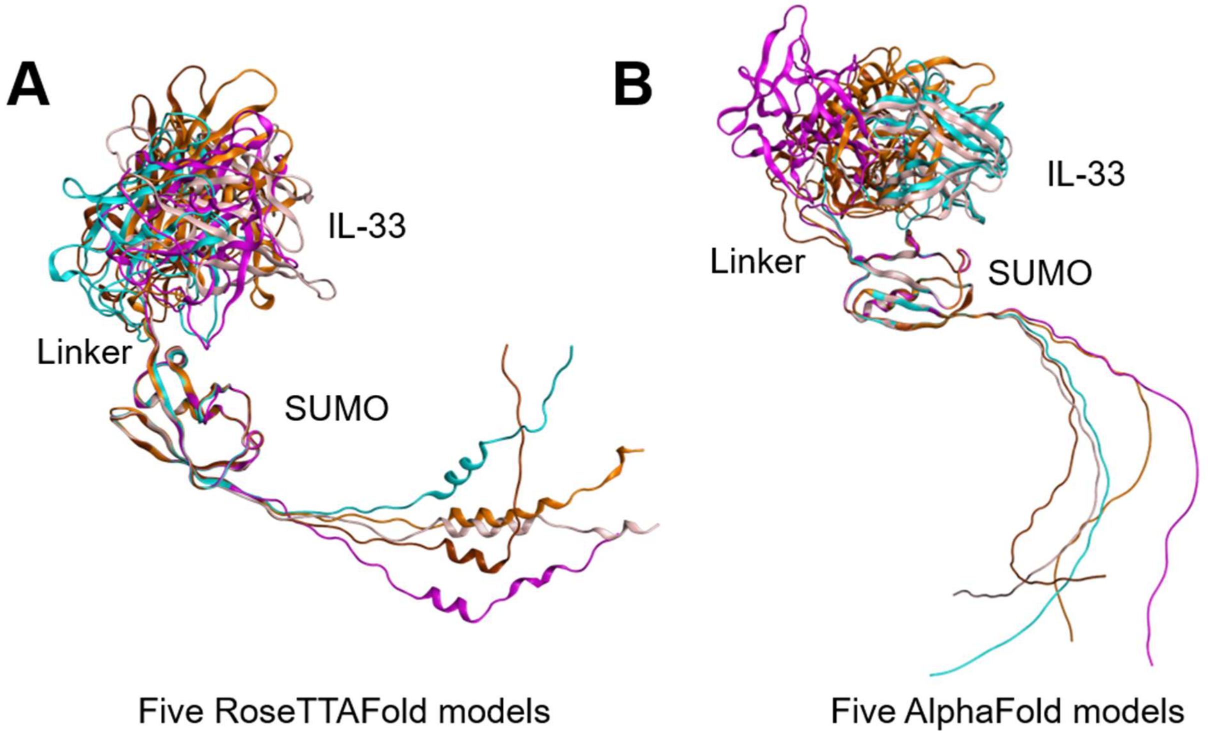

Figure S5. Structural models of SUMO-IL-33 predicted by RoseTTAFold (A) and AlphaFold2 (B).
